# Carbon risk and labor investment efficiency

**DOI:** 10.1038/s41598-025-88413-6

**Published:** 2025-02-13

**Authors:** Tingwei Luo, Fubi Luo, Xu Guo

**Affiliations:** 1 College of Business and Economics, Australian National University, Canberra, Australia; 2 School of Economics and Management, Chongqing Normal University, Chongqing, 401331 China

**Keywords:** Carbon risk, Labor investment efficiency, Environmental uncertainty, Agency problems, Managerial capacity, Climate sciences, Environmental sciences, Environmental social sciences

## Abstract

Labor capital plays a critical role in shaping enterprise competitiveness and in the allocation of macroeconomic resources, while the prominent of carbon risk significantly influences corporate labor allocation patterns. In this paper, utilizing data sampled from China’s A-share listed companies between 2012 and 2021, the impact and mechanism of corporate carbon risk on labor investment efficiency have been examined. The results reveal that carbon risk significantly inhibits labor investment efficiency. Moreover, environmental uncertainty, agency problems, and managerial ability are the primary channels through which carbon risk can affect corporate labor investment efficiency. Additional tests reveal that the impact of carbon risk on labor investment efficiency is more pronounced in firms with lower competitive positions and more severe financing constraints. The detrimental effect of carbon risk on labor investment efficiency is particularly evident in firms characterized by labor underinvestment, high labor intensity, high pollution, and non-high-tech industries. The findings of this study can provide valuable insights for policymakers to improve carbon risk management, enhance labor investment efficiency, and optimize the institutional mechanisms for factor market allocation.

## Introduction

Efficient labor allocation is considered the foundation for workforce functioning and a key prerequisite for businesses to improve the effectiveness of their human resources and generate greater value. From a macroeconomic perspective, Jung et al. (2014)^1^pointed out that labor costs, which account for approximately two-thirds of the value added to the global economy, hold significant economic importance. Due to its pronounced externalities, labor, as an essential component of enterprises’ production functions, contributes positively to factor integration, mitigates the constraints of diminishing marginal returns, and thereby fosters long-term, sustainable, and healthy economic growth^2^. At the microeconomic level, labor is recognized as a pivotal factor in corporate investment, and effective labor investment is crucial for enhancing firms’ productive capacities^3^. It also improves revenue-output efficiency, increases surpluses, strengthens stock returns^4^, and bolsters competitive advantages^5^. Consequently, labor investment efficiency emerges as a critical dimension of macroeconomic development, reflecting the effectiveness of resource allocation. Simultaneously, it represents a fundamental factor directly linked to the enhancement of corporate value. Over the past decade, China has encountered an increasingly severe aging population issue, accompanied by a gradual reduction in the demographic dividend. This demographic shift has led to a marked decline in both the quantity and quality of the labor supply, thereby eroding the traditional cost advantages of labor. The substantial contraction in the workforce presents a significant challenge to sustainable economic growth. Moreover, the current labor market allocation is plagued by issues such as gender discrimination, restrictions linked to the household registration system, and deficiencies in social security coverage, all of which impede the free mobility and equitable competition of labor. These structural challenges not only constrain the development of high-quality talent but also undermine the overall efficiency of labor resource allocation^6^. Consequently, given the current reality of a shrinking and misaligned labor supply, it is of considerable theoretical and practical importance to examine ways to optimize human capital allocation and enhance labor investment efficiency for achieving high-quality development in enterprises.

Currently, China has identified the realization of its “dual-carbon” goal as a central pillar of its economic development strategy. During the seventy-fifth session of the United Nations General Assembly, President Xi Jinping announced that China would strive to achieve peak carbon dioxide emissions by 2030 and attain carbon neutrality by 2060. This commitment was later reaffirmed at the Central Economic Work Conference, which emphasized that “achieving carbon neutrality is an intrinsic prerequisite for maintaining high-quality growth.” As China advances toward its “dual-carbon” objectives, carbon risk has emerged as a critical consideration in corporate operations. It also represents a key determinant of sustainable and high-quality economic development. For businesses to align with the “dual-carbon” agenda, it is imperative to proactively address carbon emissions and devise innovative solutions to reduce their carbon footprints. According to China’s Corporate Social Responsibility (CSR) reports, corporate carbon risk primarily encompasses regulatory, reputational, and competitive risks. To mitigate these risks, enterprises with high carbon exposure must increase their green investments, such as hiring environmentally skilled technical personnel and management talents with experience in sustainable practices, which may compel firms to enhance their labor investment efficiency. However, the increased investment required to address carbon risk can also deplete organizational resources. Furthermore, these investments might be exploited by managers as tools for self-interest, potentially resulting in underemployment and a decline in labor investment efficiency.

Carbon risk can exacerbate environmental uncertainty, amplify managerial agency problems in labor investment decision-making, and impair managers’ ability to make effective information-based decisions, thereby reducing corporate labor investment efficiency. However, existing literature on the economic consequences of carbon risk predominantly focuses on the investment efficiency of physical and financial assets, with relatively little attention paid to labor investment efficiency. Most studies explore how factors such as corporate strategy, information quality, executive characteristics, social trust, governance mechanisms, and policy frameworks influence labor investment efficiency and have overlooked the role of corporate green performance in human resource allocation. Labor, as a critical factor of production, is not only a key determinant of social productivity levels but also one of the most dynamic factors influencing corporate output and profitability. In modern economic systems, the rational allocation of labor plays an increasingly important role. Consequently, this study examines the economic consequences of carbon risk from the perspective of labor investment efficiency. The impact of carbon risk on labor investment efficiency remains underexplored, highlighting a gap that warrants further investigation. To address this gap, this study examines the relationship and underlying mechanisms between carbon risk and labor investment efficiency. By doing so, it seeks to contribute to the literature on the economic consequences of carbon risk and the determinants of labor investment efficiency. Using a research sample of A-share listed companies from 2012 to 2021, this paper integrates carbon risk and labor investment efficiency into a unified analytical framework to evaluate the impact of carbon risk on labor investment efficiency and to verify the mechanisms through which this influence occurs.

The potential marginal contributions of this study are primarily reflected in the following aspects: First, the integration of carbon risk and labor investment efficiency is examined, revealing that carbon risk exerts an inhibitory effect on labor investment efficiency. This finding enriches the literature on the impact of external environmental factors on internal corporate operations and management practices. Second, the study explores the detailed mechanisms through which carbon risk influences labor investment efficiency, thereby expanding the research landscape of corporate labor investment efficiency. Third, the study investigates enterprise heterogeneity, examining how carbon risk differently affects the labor investment efficiency of firms with varying levels of labor investment, labor intensity, pollution levels, and technological capabilities. Fourth, the paper proposes a strategy for businesses to enhance their response to carbon risk, improve labor investment efficiency, and optimize the institutional mechanisms for macro-level market allocation of factors. Additionally, the study offers policy recommendations for government support aimed at reducing carbon emissions in enterprises, thus contributing to the promotion of low-carbon development.

## Literature review and research hypotheses

### Literature review

#### Economic consequences of carbon risk

According to China’s Corporate Social Responsibility (CSR) report, corporate carbon risks mainly encompass regulatory risks, reputational risks, and competitive risks. Firstly, regulatory risk primarily refers to the risk brought about by carbon emission regulations and policies established by the government and international organizations. As governments and international organizations continue to strengthen their oversight of carbon emissions, policies such as carbon taxes, carbon markets, and emission restrictions have been implemented. Enterprises must comply with these regulations to avoid the risk of fines or legal litigation. Furthermore, policy uncertainty exacerbates risk for firms, as they struggle to predict future changes in carbon-related policies. Secondly, reputational risk refers to the significant impact of a company’s carbon emissions and environmental performance on its public image and brand reputation. Excessive reliance on fossil fuels, high levels of emissions, or failure to adopt environmental measures may cause firms to be perceived as environmentally irresponsible, thereby damaging their brand reputation. Such negative perceptions could lead to consumer boycotts, ultimately harming the company’s market share. Finally, competitive risk arises from the need for firms to adapt to climate change by altering their business models. Due to climate change, enterprises will face challenges in developing new low-carbon technologies and producing low-carbon products to address the competitive threats of new markets and consumer demands.

Research on the economic consequences of carbon risk can be broadly categorized into three primary areas: the effects of carbon risk on corporate performance, the influence of carbon risk on corporate investment decisions, and the impact of carbon risk on corporate financial activities.

Studies examining the impact of carbon risk on corporate performance can be categorized into two main areas: its effects on financial performance and its influence on corporate value. Regarding the relationship between carbon risk and corporate financial performance, scholars have proposed two contrasting perspectives. The first perspective suggests that high-carbon-emitting companies face significant compliance and governance costs, which deplete their cash flow, increase their financial burdens, and ultimately lead to a reduction in financial performance^7^. A second group of scholars argue that, in the short term, the investments made by enterprises to enhance their carbon performance will not be recognized by the market and investors. These stakeholders often view such investments as an “expense” rather than an “investment”, which is ultimately reflected negatively in the enterprises’ financial performance^8^. The second perspective posits that companies adopt a proactive approach to addressing carbon risk by reducing emissions through technological innovation and the development of green and low-carbon technologies^9^. This will reduce fines arising from carbon emissions and the financial burden on businesses^10^. Furthermore, such a proactive attitude not only aids in building a positive corporate image but also enhances reputation and competitiveness, ultimately achieving a win-win scenario of reduced carbon emissions and improved financial performance^11^. In the research process of carbon risk on corporate value, the existing literature concurs that high carbon emissions adversely affect corporate value, indicating a negative correlation between carbon emission levels and company value. Choi et al. (2020)^12^, using data from firms across 28 countries, examined the value relevance of carbon emissions under varying institutional, regulatory, and cultural contexts. Their findings reveal that, in scenarios where firms voluntarily disclose carbon information, higher carbon emission levels are negatively associated with firm value. This negative relationship is particularly pronounced in countries with more stringent carbon regulations.

Research on the impact of carbon risk on corporate investment has primarily focused on its effects on corporate mergers and acquisitions (M&A) behavior and innovation activities. Existing literature suggests that high-carbon-emitting enterprises tend to choose companies in countries or regions with weaker environmental regulations for cross-border M&A to diversify carbon risk^13^. However, this approach is not effective in the long term. As high-energy-consuming industries develop in the countries of the acquired firms, the carbon emissions of the host countries will hinder the performance of cross-border M&A^14^. In contrast, reducing environmental costs through technological innovation presents a more sustainable approach than relying on cross-border M&A^15^. Most studies examining the impact of carbon risk on corporate innovation align with Porter’s hypothesis, which posits that environmental regulations can drive firms to innovate. For instance, Zhou et al. (2019)^9^argued that a firm’s awareness of carbon risk is positively correlated with its investment in innovation. Similarly, Zhang et al. (2022)^16^ suggested that carbon emissions trading pilot policies can enable enterprises to allocate more funds to innovation by reducing debt financing costs.

Research on the impact of carbon risk on corporate finance generally approaches from two perspectives: financial leverage and the cost of capital. Regarding financial leverage, scholars present two primary views regarding the relationship between carbon risk and financial leverage. The first view posits that carbon risk reduces corporate financial leverage. Proponents of this perspective argue that, according to the capital structure trade-off theory, when the increase in debt financing costs due to carbon risk exceeds the tax shield benefits, firms will lower their debt financing proportion. As a result, firms can lower financial leverage by increasing financial distress risk. Nuyen and Phan (2020)^17^reached the same conclusion by analyzing the impact of increasing carbon risk on the corporate capital structure after the signing of the Kyoto Protocol in Australia. In contrast, the second view posits that heightened carbon risk may increase financial leverage. Scholars supporting this perspective, drawing on Porter’s hypothesis, suggest that firms may proactively innovate to manage carbon risk. However, technological innovation typically requires significant capital, which may compel firms to access external capital markets, thereby increasing debt financing and financial leverage^18^.In terms of the cost of capital, scholars examine both the cost of debt and the cost of equity. The prevailing consensus is that elevated carbon risk raises debt financing costs, as creditors incorporate carbon risk into their credit assessments. High carbon risk increases uncertainty in a firm’s cash flows and raises the likelihood of debt default, prompting creditors to demand higher returns. This, in turn, raises the cost of debt capital^8^. Regarding the cost of equity capital, existing literature indicates that investors respond to high carbon risk by demanding higher expected stock returns to compensate for the increased risk. This behavior leads to an elevated cost of equity capital^19^.

#### Factors affecting the efficiency of labor investment

Labor investment refers to a firm’s allocation of resources toward human capital, while labor investment efficiency denotes the alignment between the actual labor employed and the optimal labor required for production and operational activities. This efficiency is influenced by firm strategy^20^, information quality^21^, and executive characteristics^22^, along with factors such as social trust, governance mechanisms, and policy regimes^23,24^. Effective labor investment ensures that labor input remains at the optimal level, eliminating redundancy and underemployment, thereby maximizing returns on labor investment.

The existing literature has relatively underexplored the impact of external environmental factors on the efficiency of labor investment, with only limited studies addressing the macro-legal and policy environment. For instance, Kong et al. (2020)^25^considered the external policy impact of the Labor Contract Law to raise labor costs and focus on labor-intensive industries. Their findings indicate that labor protection policies negatively impact corporate labor investment efficiency, particularly in state-owned enterprises. Similarly, Luo et al. (2020)^26^investigated how political uncertainty influences labor investment efficiency at the macro level, discovering that political uncertainty exacerbates information asymmetry, ultimately reducing labor investment efficiency. Moreover, the negative impact of political uncertainty is more significant when newly appointed government officials are older, the firm is state-owned, belongs to a politically sensitive industry, or operates in a region with stringent labor protections. Conversely, this adverse effect is less pronounced in regions with weak government intervention or those that have experienced anti-corruption campaigns. Kong et al. (2018)^27^ also explored the impact of the political environment on firms’ labor investment efficiency from the perspective of local officials’ promotions.

Existing research has extensively examined carbon risk and labor investment efficiency, offering valuable insights that serve as a reference for this paper. However, notable gaps remain. While a substantial body of literature investigates the economic effects of carbon risk-such as its impact on corporate investment, financing, and performance-and explores factors influencing labor investment efficiency, including corporate strategy, information quality, executive characteristics, social trust, governance mechanisms, and policy frameworks, limited attention has been devoted to the specific relationship between carbon risk and corporate labor investment efficiency. The mechanisms and pathways through which carbon risk affects labor investment efficiency remain underexplored. Furthermore, existing studies predominantly emphasize capital investment efficiency, with relatively little focus on labor investment efficiency, underscoring the need for further investigation in this area.

This study makes several marginal contributions: First, it integrates analyses of carbon risk and labor investment efficiency from the perspective of labor investment efficiency, revealing the inhibitory effect of carbon risk on labor investment efficiency and enriching the literature on the impact of the external environment on internal corporate operations and management behaviors. Second, the study explores the detailed mechanisms and effects of carbon risk on labor investment efficiency, broadening the research landscape of enterprise labor investment efficiency. Third, it conducts a comprehensive heterogeneity analysis, examining differences in the impact of carbon risk on labor investment efficiency across various types of labor investment, labor intensity, pollution degree, and technology level. Fourth, from a practical perspective, the study offers actionable strategies for enterprises to enhance their responses to carbon risk. It proposes ways to improve labor investment efficiency and optimize institutional mechanisms for the market-oriented allocation of macroeconomic factors. Additionally, it provides policy insights for government support of enterprises’ low-carbon emission reduction efforts.

### Research hypotheses

#### Carbon risk and labor investment efficiency

Labor investment efficiency is a critical indicator of resource allocation efficiency, enterprise management capability, innovation performance, and overall development quality. It is influenced not only by a company’s strategic direction and managerial practices but also by the external development environment and regulatory oversight. Higher carbon risk means higher regulatory risk, reputation risk, and competitive risk for enterprises, which will reduce labor investment efficiency and hinder their long-term business growth.

First, enterprises undertake green governance and environmental protection investments to mitigate carbon risk, but such investments do not necessarily yield direct economic benefits. They may deplete the company’s resources^28^, crowding out labor investment and thereby reducing labor investment efficiency. For instance, efforts to establish a strong reputation as a green benchmark by reducing carbon emissions often result in higher environmental management costs, erosion of production and operational profits, strained cash flows, and resource shortages. To weaken these adverse effects, enterprises may choose to streamline their employees, excessively fire them, or reduce their benefits, which can lead to employee disengagement and lower labor investment efficiency^6^. Second, high carbon risk often signals poor corporate governance and weak managerial capabilities to external stakeholders, damaging an enterprise’s reputation and eroding investor trust and support. This lack of confidence raises financing costs and exacerbates financial constraints, leaving firms with insufficient resources to invest in human capital or cultivate high-tech, innovative talent, thereby further reducing labor investment efficiency. Third, enterprises with high carbon risk are subject to stringent environmental regulations. Compliance with carbon emission standards necessitates low-carbon transformation and the production of green products, which heightens competitive risks in the product market. Under uncertain business conditions, firms may defensively dismiss employees to reduce costs. Simultaneously, core employees may proactively resign in search of better opportunities for their long-term career development. These dynamics contribute to underemployment and exacerbate the decline in labor investment efficiency.

In summary, the following hypothesis is proposed:

H1: Carbon risk diminishes the efficiency of enterprises’ labor investment.

#### Analysis of the mechanism of carbon risk on labor investment efficiency

The implementation of China’s “dual-carbon” strategy may pose numerous challenges for companies operating in high-carbon-risk environments. These companies may need to adopt preventative environmental measures to accommodate evolving regulations and market requirements, which may hurt labor investment efficiency. Meanwhile, in modern enterprise operation practice, labor investment may prioritize superficial objectives over core goals and be inefficient due to the existence of principal-agent and information asymmetry problems^29^. Therefore, the following analysis examines the mechanism through which carbon risk affects labor investment efficiency from multiple perspectives based on resource-based view theory, principal-agent theory and information asymmetry theory.

(1) According to the resource-based view, enterprises must depend on a particular environment for survival, while this environment simultaneously imposes constraints on the firm’s operations. As a result, managers are required to formulate distinct business strategies and operational decisions based on the specific characteristics of the environment in which the enterprise operates. Carbon risk amplifies environmental uncertainty, thereby inhibiting firms’ labor investment efficiency. Environmental uncertainty is used to measure the degree of environmental changes in a firm’s operations, specifically caused by the unpredictable behavior of customers, suppliers, competitors, and regulatory groups. Their behavior is random and difficult to predict^30,31^. From both political economy and survival perspectives, the reduction of greenhouse gas (GHG) emissions is an irreversible policy^32^. Carbon risk arises from uncertainty in firms’ expectations and regulatory activity during the transition to a low-carbon economy^19^. Most regulatory measures to restrict carbon emissions, such as carbon taxes, cap-and-trade schemes, and emission limits, aim to address environmental challenges. However, significant uncertainties remain regarding the timing, implementation, and enforcement of these policies. For example, in the case of a carbon tax, the high degree of uncertainty regarding carbon emission prices and the difficulty in precisely estimating future carbon pricing trends exacerbate environmental uncertainty for firms. Research has shown that the uncertainty generated by policy regulation can alter a firm’s market capability, future cash flow, and performance due to factors such as financial constraints, competition levels, and industry characteristics^33,34^. Therefore, enterprises exposed to higher levels of carbon risk experience greater environmental unpredictability.

Environmental uncertainty makes it more difficult for stakeholders to evaluate and monitor managers’ decisions and firm performance. Additionally, the increased volatility and unpredictability of firms’ profitability in an uncertain environment, coupled with greater information asymmetry between firms and the external market^35^, make it more challenging for managers to make effective decisions based on market information^36^. Consequently, labor investment decisions are more likely to deviate from the optimal level, inhibiting the firm’s labor investment efficiency. Bu and Sun (2020)^37^, using data from China’s A-share listed companies in non-financial industries, demonstrated that environmental uncertainty suppresses corporate labor investment efficiency.

In summary, the following hypothesis is proposed:

H2: Carbon risk dampens labor investment efficiency by exacerbating environmental uncertainty.

(2) According to the principal-agent theory, the ownership and control of an enterprise are separated. Shareholders and managers are limited rationality “economic men”, often leading to conflicts of interest due to misaligned objectives. When there is a serious agency conflict, enterprise management tends to take opportunistic behavior to achieve personal self-interest or build a “personal empire”, which undermines the improvement of labor investment efficiency within the organization. Carbon risk exacerbates these managerial agency problems in labor investment decisions, thereby inhibiting firms’ labor investment efficiency. On the one hand, high-carbon-risk firms face greater environmental pressure, tending to engage in opportunistic behavior^35,38^. Meanwhile, carbon risk provides an excuse for managers’ inability to meet performance goals, allowing them to obscure self-interested behaviors under the guise of environmental volatility. On the other hand, carbon risk increases the difficulty of assessing a firm’s business performance, making it harder for stakeholders, including shareholders, to effectively monitor and scrutinize corporate investment decisions. This creates opportunities for managerial laziness and on-the-job consumption^39^, resulting in decisions that deviate from the optimal level.

In terms of labor investment decisions, labor exhibits greater mobility and reversibility than other factors, giving managers more discretion in labor investment decisions. Consequently, firms are more likely to use labor allocation adjustments as a preliminary step when developing their overall investment strategy^40^. Under high carbon risk, managers may retain excessive redundant labor to build a “business empire” due to self-interested incentives and inadequate stakeholder monitoring, or conversely, maintain low labor employment levels for reputational or risk-averse reasons. These actions cause labor investment to deviate from the optimal level, thereby reducing labor investment efficiency.

In summary, the following hypothesis is proposed:

H3: Carbon risk inhibits labor investment efficiency by exacerbating agency problems.

(3) According to information asymmetry theory, in market economic activities, there are differences in the degree of understanding of relevant information among traders. The party with sufficient information holds an advantageous position, while the party with limited information is at a relative disadvantage in the transaction. Carbon risk weakens managers’ ability to make effective information-based decisions, thereby restraining corporate labor investment efficiency. In the process of enterprise investment decision-making—whether during pre-investment evaluation, mid-investment monitoring, or post-investment operations—managers need to make corresponding decisions based on a large amount of environmental information and adjust their resource allocation behaviors in response to environmental fluctuations. However, the environmental uncertainty induced by carbon risk increases the cost and difficulty for firms to obtain necessary decision-making information from the market, resulting in a higher risk of impaired managerial decision-making effectiveness^41^. This, in turn, affects the implementation of strategic objectives and the effectiveness of decision-making in resource allocation. Therefore, carbon risk increases information asymmetry between enterprises and their external environment, limiting managers’ ability to acquire and utilize relevant information and ultimately reducing the efficiency of their decision-making.

When carbon risk is high, corporate surpluses face greater volatility and unpredictability, the degree of information asymmetry between enterprises and the external market increases, and managers’ capacity to make reasonable and effective decisions based on information declines. Although the labor factor has a lower adjustment cost compared to other factors^1^, managers are required to make timely and accurate adjustments to labor investment based on environmental fluctuations. Additionally, because labor costs are variable, labor budgets are typically based on sales budgets. A reasonable estimation of labor investment decisions must consider relevant information, such as expected market sales budgets. However, carbon risk exacerbates the difficulty and unpredictability of such assessments. Consequently, managers face significant challenges in obtaining reliable information for decision-making in labor investment, increasing the likelihood of deviations from optimal labor investment levels and ultimately inhibiting labor investment efficiency.

In summary, the following hypothesis is proposed:

H4: Carbon risk inhibits labor investment efficiency by reducing managerial capacity.

## Research design

### Definition of variables

#### Efficiency of labor investment

Labor investment efficiency refers to the extent to which a firm’s labor investment deviates from the optimal level. Existing literature typically measures labor investment through the net change in employee numbers, expressed as the ratio of the change in the number of employees in the current year to the number in the previous year. The greater the deviation of the actual level of labor investment from the optimal (expected) level, the larger the difference, and the more inefficient the firm’s labor investment is.

Drawing on Jung et al. (2014)^1^, which is first based on Pinnuck and Lillis (2007)^40^_,_ this study measures firms’ net employment by the percentage change in the number of employees hired. Several other relevant economic variables are then regressed according to the rate of change in the number of employees hired, after controlling for industry effects. The regression yields a series of abnormal labor investment efficiencies. Specifically, the following model 1 is used for the regression.

1 $$\begin{gathered} Net\_Hir{e_{it}}={\beta _0}+{\beta _1}Sale{\text{s}}\_Growt{h_{it - 1}}+{\beta _2}Sales\_Growt{h_{it}}+{\beta _3}RO{A_{it - 1}}+{\beta _4}RO{A_{it}} \\ +{\beta _5}\Delta RO{A_{it}}+{\beta _6}Size\_{R_{it - 1}}+{\beta _7}Quic{k_{it - 1}}+{\beta _8}Quic{k_{it}}+{\beta _9}\Delta Quic{k_{it}} \\ +{\beta _{10}}Le{v_{it - 1}}+{\beta _{11}}Lossbin{1_{it - 1}}+{\beta _{12}}Lossbin{2_{it - 1}}+{\beta _{13}}Lossbin{3_{it - 1}} \\ +{\beta _{14}}Lossbin{4_{it - 1}}+{\beta _{15}}Lossbin{5_{it - 1}}+{\varepsilon _{it}} \\ \end{gathered}$$

In this model, *Net_Hire* denotes the percentage change in the number of employees, that is, the rate of change in the ratio of the total number of employees in the year to the total market capitalization of the individual stocks. *Sales_Growth* denotes the difference in operating income between the current year and the previous year. *ROA* is the return on assets. *Size_R* is the percentile ranking of the total market capitalization of the individual stocks for the year. *Quick* is the quick ratio. Lev denotes the long-term debt ratio. *Lossbinx* is a dummy variable for five intervals, each with a length of 0.005, for the previous year’s *ROA* in the range of [−0.025, 0]. *Lossbin1* takes the value of 1 when *ROA* falls into [−0.005, 0] and 0 otherwise. *Lossbin2* takes the value of 1 when *ROA* falls into [−0.010, −0.005] and 0 otherwise, and so on.

The residual from model 1, denoted as *Abresid*, serves as a measure of labor investment efficiency. This indicator is an inverse measure, meaning that the larger the absolute value of the residual, the greater the deviation of the firm’s net employment level from the expected value, indicating a lower labor investment efficiency. If the actual rate of change in personnel exceeds the expected rate estimated by the model, it is classified as redundant employment (*Mresid*). Conversely, if the actual rate of change in personnel is less than the expected rate, it is classified as under-employment *(Lresid*). Both redundant employment and underemployment are indicative of labor investment inefficiency.

#### Carbon risk

Most international scholars use the ratio of carbon emissions to operating revenue as a proxy indicator for carbon risk. However, due to the incomplete disclosure of carbon emission data in China’s Carbon Disclosure Project (CDP) and the lack of comprehensive publication of carbon emission data for listed companies, this study follows the methodology of Wang et al. (2022)^42^ and calculates enterprise carbon emissions using the formula: Carbon Emissions = Combustion and Fugitive Emissions + Production Process Emissions + Waste Emissions + Emissions from Land-Use Changes (e.g., conversion of forests to industrial land). The data for these calculations are manually collected from various sources, including annual reports of listed companies, Corporate Social Responsibility (CSR) reports, company websites, and the websites of environmental regulatory authorities. The ratio of carbon emissions to operating revenue is then used to measure the carbon risk level (*Risk*), specifically expressed as: Carbon Risk Level of Enterprises = Enterprise Carbon Emissions/Enterprise Operating Income. A higher value of this indicator signifies a greater level of carbon risk for the enterprise.

#### Control variables

Referring to studies in the existing literature^1,37^, the following control variables are introduced: the size of the enterprise *(Size)*, years of establishment (*Age)*, asset leverage ratio *(Lev*), return on net assets (*Roa)*, cash flow ratio (*Cfo*), book-to-market ratio (*Bm*), management shareholding ratio (*Mhold*), and combining the CEO and Chairman (*Dual*). In addition, the effects of firm industry and year are controlled. The variables are defined as shown in Table 1.

**Table 1:** List of variable definitions.

Variable code	variable name	variable measure
*Abresid*	efficiency of labor investment	Absolute value of model 1 residuals
*Risk*	carbon risk	Carbon emissions/Operating income
*Size*	the size of the enterprise	The logarithm of total assets
*Age*	years of enterprise	Year of observation - year of establishment
*Lev*	asset leverage ratio	Total liabilities to total assets
*Roa*	return on assets	Net profit to total assets
*Cfo*	cash flow ratio	Net cash flows from operating activities to total assets
*Bm*	book-to-market ratio	Total assets to market capitalization
*Mhold*	management shareholding	Number of shares held by directors and supervisors to total shares
*Dual*	combining the CEO and Chairman	1 for CEO as chairman, and 0 for others

### Sample selection and data sources

Given that the carbon emissions trading pilot was launched in November 2011, this study selects A-share listed companies from 2012 to 2021 as the research sample and excludes the following samples: (1) companies with fewer than 30 employees; (2) companies in the insurance and financial industries; (3) companies with missing data; and (4) companies with outliers. A total of 7234 samples are obtained. In addition, all continuous variables in this study are winsorized at the top and bottom 1% to mitigate the influence of extreme values.

Labor investment efficiency is calculated based on model 1  presented in the article, while carbon risk is measured using the ratio of carbon emissions to operating income, following the methodology of Wang et al. (2022)^42^. Carbon emissions are determined using the formula: Carbon Emissions = Combustion and Fugitive Emissions + Production Process Emissions + Waste Emissions + Emissions from Land-Use Changes (e.g., conversion of forests to industrial land).

The data used for these calculations are derived from multiple sources, including the annual reports of listed companies, Corporate Social Responsibility (CSR) reports, company websites, and environmental regulatory websites. Additional data are obtained from the CSMAR database: https://www.csmar.com/channels/31.html.

### Modeling

To test the hypotheses of this paper, we developed the following regression model:

2 $$Abresi{d_{i,t}}={\alpha _0}+{\alpha _1}Ris{k_{i,t}}+Control{s_{i,t}}+\sum {Year} +\sum {Indcd} +{\varepsilon _{i,t}}$$

*Abresid* is an inverse indicator. If the regression coefficient of *Risk* in the model 2 is significantly greater than 0, it indicates that the higher the carbon risk of the enterprise, the lower the efficiency of its labor investment, thus proving hypothesis H1. *Year* represents the year dummy variable and *Indcd* is the industry dummy variable.

## Analysis of empirical results

### Descriptive statistics

Table 2 presents the descriptive statistics of the main variables. The mean (median) of Labor Investment Efficiency (*Abresid*) is 0.238 (0.174), with a standard deviation of 0.25, which aligns with the findings reported in the existing literature. The mean (median) of Carbon Risk (*Risk*) is 0.455 (0.432), with a standard deviation of 0.162, indicating differences in carbon risk across firms.

**Table 2:** Results of descriptive statistics.

Variant	*N*	Mean	p50	SD	Min	Max	Range
*Abresid*	7234	0.238	0.174	0.250	0.000	1.569	1.569
*Risk*	7234	0.455	0.432	0.162	0.158	1.115	0.956
*Size*	7234	22.611	22.352	1.436	20.186	27.021	6.835
*Age*	7234	18.495	18.000	5.352	6.000	32.000	26.000
*Lev*	7234	0.457	0.460	0.198	0.066	0.887	0.821
*Roa*	7234	0.035	0.033	0.052	−0.187	0.177	0.346
*Cfo*	7234	0.049	0.048	0.062	−0.123	0.223	0.346
*Bm*	7234	0.641	0.638	0.260	0.133	1.211	1.079
*Mhold*	7234	0.111	0.002	0.1775	0.000	0.636	0.636
*Dual*	7234	0.248	0.000	0.432	0.000	1.000	1.000

### Benchmark regression

Table 3 presents the regression results of model  2. Column (2) further controls for industry and year effects based on the relevant factors controlled for in column (1). As shown in Table 3, the coefficient between carbon risk (*Risk*) and labor investment efficiency (*Abresid*) is significantly positive at the 1% level. This suggests that carbon risk negatively impacts labor investment efficiency, thereby confirming H1.

**Table 3:** Benchmark regression results.

Variant	(1)	(2)
	Abresid	Abresid
*Risk*	0.090^***^	0.076^***^
	(4.901)	(4.094)
*Size*	−0.000	0.005^*^
	(−0.070)	(1.725)
*Age*	−0.001^*^	−0.000
	(−1.900)	(−0.664)
*Lev*	0.066^***^	0.061^***^
	(3.247)	(2.987)
*Roa*	0.130^*^	−0.013
	(1.882)	(−0.186)
*Cfo*	−0.162^***^	−0.103^*^
	(−3.066)	(−1.932)
*Bm*	−0.033^**^	−0.106^***^
	(−2.186)	(−6.484)
*Mhold*	0.030	0.052^***^
	(1.535)	(2.645)
*Dual*	0.003	0.004
	(0.395)	(0.561)
*constant*	0.212^***^	0.230^***^
	(3.346)	(3.355)
*N*	7234	7234
*R* ^*2*^	0.009	0.064
*Year*	NO	YES
*Indcd*	NO	YES

Note: t-values are in parentheses; ^*^, ^**^, and^***^ indicate 10%, 5% and 1% significance levels, respectively. Same as below.

## Robustness tests

### Endogenous issues

#### Instrumental variables regression

To address the issue of omitted variables and potential two-way causality, this study employs the instrumental variable (IV) approach, using the industry mean value of carbon risk (*IV_R1*) and the number of environmental regulations in each province (*IV_R2*) as instruments. Generally, the industry average of carbon risk and the number of environmental regulations in each province are closely related to corporate carbon risk, satisfying the correlation hypothesis. Furthermore, the industry mean of carbon risk is not correlated with the random disturbance term, satisfying the exogeneity assumption. The industry mean of carbon risk can affect labor investment efficiency only by influencing corporate carbon risk, not through other pathways. The number of environmental regulations in each province is also uncorrelated with the random perturbation term, and cannot directly affect the efficiency of the firm’s labor investment, thereby satisfying the exogeneity assumption.

The results are presented in column (1) and column (2) of Table 4. The first-stage results reveal that the coefficient of the regression of the industry average of carbon risk (*IV_R1*) on carbon risk (*Risk*) is 0.092, and the coefficient of the regression of the number of environmental regulations in each province (*IV_R2*) on carbon risk (*Risk*) is 0.083. These results indicate that both the industry average of carbon risk and the number of environmental regulations in each province are significantly and positively correlated with corporate carbon risk. Furthermore, the F-statistic in the first stage exceeds 10, rejecting the hypothesis that the instrumental variable is weak. In the second stage, the regression coefficient of carbon risk is significantly positive at the 1% level, indicating that carbon risk negatively affects labor investment efficiency of enterprises.

#### PSM test

To address potential sample selection bias, this study employs the propensity score matching (PSM) method for testing. The core idea of PSM is to screen out matched samples with identical characteristics from the control and experimental groups, ensuring that the only difference between the two groups is whether they are in the experimental state. This approach achieves results comparable to those of a randomized experiment. The original intention of PSM is to balance certain factors (interfering independent and dependent variables) between the treatment and control groups, mitigating their interference on the results and thus alleviating the endogeneity problem. Initially, all control variables from the previous analysis are selected as covariates, with industry and time effects also controlled for. The 1:1 nearest-neighbor matching method is used to identify the control group matched with the experimental group. The experimental group consists of firms with labor investment efficiency above the annual industry median, while the remaining firms constitute the control group. After matching, there is no significant difference between the experimental and control groups for each control variable, better passing the test of sample balance. Subsequently, regression analyses are conducted on this matched sample. The results are shown in column (3) of Table 4. The regression coefficient of carbon risk (*Risk*) remains significantly positive at the 1% level, confirming that the primary findings are robust.

#### Controlling for individual fixed effects

To address potential bias arising from omitted variables, the study re-examines the model by incorporating firm-level fixed effects. The regression results shown in column (4) of Table 4 indicate that carbon risk (*Risk*) remains positively correlated with the labor investment efficiency indicator (*Abresid*) at the 1% level. This demonstrates that the findings remain unchanged after controlling for omitted variables using the fixed effects model.

**Table 4:** Endogeneity test regression results.

Variant	(1)	(2)	(3)	(4)
	Instrumental variable approach		PSM test	Fixed effect
	phase I	phase II		
	*Risk*	*Abresid*	*Abresid*	*Abresid*
*Risk*		0.088^***^	0.071^***^	0.075^***^
		(4.251)	(3.156)	(3.374)
*IV_R1*	0.092^***^			
	(2.726)			
*IV_R2*	0.083^**^			
	(2.563)			
*constant*	0.534^***^	0.239^***^	0.399^***^	−0.670^**^
	(9.981)	(3.470)	(2.875)	(−2.211)
N	7234	7234	7234	7234
R^2^	0.098	-	0.059	0.066
F-value	11.32	-	-	-
Controls	Yes	Yes	Yes	Yes
*Year*	Yes	Yes	Yes	Yes
*Indcd*	Yes	Yes	Yes	Yes

### Other robustness tests

#### Replace dependent variable

The previous section measured the efficiency of labor investment in enterprises based on the number of employees they employed. To further test the robustness of the results, alternative approaches are employed to re-measure labor investment efficiency in enterprises.

(1)Drawing on Jung et al. (2014)^1^and Chen et al. (2018)^43^, the rate of change of employees (i.e., the percentage change in the total number of employees in listed companies) is used to replace the explanatory variable (*Net_Hire*) in the model 1, and the labor investment efficiency *Abresid_Count* is re-estimated for testing. The regression results are shown in column (1) of Table 5, indicating that the findings remain unchanged.

(2) Since firms’ investment in labor factors is reflected not only in changes in the number of employees but also in the level of employee payments, drawing on Chen et al. (2018)^43^and Chu and Fang (2020)^44^, the level of employee compensation (calculated as cash paid to and for employees divided by total assets) is used to replace the explanatory variable (*Net_Hire*) in the model 1. The labor investment efficiency, now denoted as *Abresid_Cash*, is re-estimated for testing. The regression results, shown in column (2) of Table 5, indicate that the findings are robust and unchanged.

#### Replace independent variable

Following Bolton and Kacperczyk (2021)^45^, the variable *G_Carbon* is constructed to measure corporate carbon risk using the growth rate of carbon emissions of listed firms. Column (3) in Table 5 shows the empirical results after replacement, and the regression coefficient of *G_Carbon* remains significantly positive, indicating that the previous conclusion still holds.

#### Use of the change model

To further observe the incremental impact of carbon risk on firms’ labor investment efficiency, this paper uses the Change model, with all variables differenced to the first order. As shown in column (4) of Table 5, the regression coefficient of incremental carbon risk (*dRisk*) is 0.059, which is significantly positive at the 5% level. This indicates that in the incremental dimension, carbon risk still has an inhibitory effect on the labor investment efficiency of enterprises, and the results of the previous study are reliable.

**Table 5:** Other robustness tests.

Variant	(1)	(2)	(3)	(4)
	Replace dependent variable		Replace independent variable	Change model
	Abresid_Count	Abresid_Cash	Abresid	dAbresid
*Risk*	0.060^***^	0.003^**^		
	(2.591)	(2.362)		
*G_Carbon*			0.019^***^	
			(2.745)	
*dRisk*				0.059^**^
				(2.578)
*constant*	0.085^***^	0.073^**^	0.220^**^	−0.087^**^
	(2.836)	(2.559)	(2.516)	(−2.192)
N	5429	6649	5443	5456
R^2^	0.028	0.023	0.063	0.047
Controls	Yes	Yes	Yes	Yes
*Year*	Yes	Yes	Yes	Yes
*Indcd*	Yes	Yes	Yes	Yes

›

## Further analysis

### Analysis of impact mechanisms

The previous section verified the inhibitory effect of corporate carbon risk on labor investment efficiency. Here, the mechanism between the two will be empirically tested based on the previous section’s theoretical analysis. The theoretical analysis mentions that carbon risk leads to increased environmental uncertainty. At the same time, carbon risk increases the unpredictability and difficulty in assessing enterprise operations, exacerbating the agency problem between management and shareholders. This provides space and ability for managers to engage in inefficient labor investment, carbon risk will inhibit labor investment efficiency through increased agency costs. Additionally, carbon risk exposes corporate surplus to greater volatility and unpredictability and increases the degree of information asymmetry between the enterprise and the external market. As a result, the ability of managers to make rational and effective decisions based on information declines, thus leading to a reduction in labor investment efficiency.

To test the influence mechanism, model 3 was constructed:

3 $$M{V_{i,t}}={\beta _0}+{\beta _1}Ris{k_{i,t}}+Control{s_{i,t}}+\sum {Year} +\sum {Indcd} +{\varepsilon _{i,t}}$$

*MV* is the mediating variable, representing environmental uncertainty (*Eu*), agency problem (*Mfee*), and managerial capacity (*Ma*), respectively. The definitions of the other variables remain consistent with those in model 2. Specifically, the agency problem (*Mfee*) is measured using the management expense ratio; managerial capability (*Ma*) refers to the study of Demerjian et al. (2012)^46^ and a two-stage model combining Data Envelopment Analysis (DEA) and Tobit model is used to measure managerial competence; Environmental uncertainty (*Eu*) refers to the degree of changes and fluctuations faced by a firm in terms of customer markets, consumer choice preferences, and industry technology that are unpredictable and beyond the ability to control^47^. Environmental uncertainty is measured as the standard deviation of firms’ operating income^48^, with the effects of growth and industry factors further removed^35^. The following regression model was first constructed using operating income data for the past five years for each sample firm.

4 $$\operatorname{Re} venue={\alpha _0}+{\alpha _1}Year+\varepsilon$$

In model 4, *Revenue* represents operating income; *Year* is a variable representing the year, taking the value of 5 if the regression sample is the current year, 4 if it is the first year in the past, and so on. The residuals obtained from this model measure operating income excluding growth. The ratio of the standard deviation of the residuals over the past five years to the mean is used to obtain environmental uncertainty before removing the industry factor. Next, environmental uncertainty before industry exclusion is used to obtain the environmental uncertainty (*Eu*) referred to in this paper by dividing it by the industry median for the same year.

The regression results for the mechanism test are shown in Table 6. Column (1) shows the relationship between carbon risk and environmental uncertainty, and the coefficient of *Risk* is significantly positive at the 1% level, which indicates that carbon risk exacerbates environmental uncertainty and thus inhibits the labor investment efficiency of enterprises, this supports H2. Column (2) shows the results of the relationship between carbon risk and firms’ agency problems, and the regression coefficient of carbon risk (*Risk*) is significantly positive, indicating that carbon risk exacerbates firms’ agency problems and thus inhibits firms’ labor investment efficiency, confirming H3. The regression coefficient of carbon risk (*Risk*) in column (3) is significantly negative, indicating that carbon risk inhibits the ability of managers to perform, reduces their ability to make effective business decisions based on relevant information, and thus inhibits labor investment efficiency, this supports H4.

**Table 6:** Regression results for mediated effects.

Variant	(1)	(2)	(3)
	Eu	Mfee	Ma
*Risk*	0.493^***^	0.006^*^	−0.020^***^
	(7.050)	(1.681)	(2.734)
*constant*	0.406	0.474^***^	0.124^**^
	(0.927)	(22.654)	(2.319)
N	7234	7234	7234
R^2^	0.029	0.233	0.203
Controls	Yes	Yes	Yes
*Year*	Yes	Yes	Yes
*Indcd*	Yes	Yes	Yes

### Moderating effects

To test the moderating effects of industry competition and financing constraints, this study constructs model 5:

5 $$Abresi{d_{i,t}}={\alpha _0}+\alpha Ris{k_{i,t}}+{\alpha _3}Risk \times RE+Control{s_{i,t}}+\sum {Year+\sum {Indcd+{\varepsilon _{i,t}}} }$$

Where *RE* is the moderator variable, representing the degree of industry competition (*HHI*) and financing constraints (*SA*), respectively, *Risk × RE* is the interaction term, and the meanings of the other variables are the same as in model 2.

#### Impact of industry competition

Firms with a relatively strong competitive position in the product market possess greater resilience to cost fluctuations, changes in consumer demand, and market shocks^49^. On the one hand, a higher competitive position can reduce the business risk brought about by the increased bargaining power of customers. In comparison, firms with a lower competitive position in the industry, due to their relatively narrow marginal returns, will experience a greater impact on their business operations. Their decision-making is also more significantly influenced by higher carbon risks. On the other hand, a strong competitive position serves as a valuable protective mechanism, providing managers with more comprehensive information sets to make effective decisions regarding the allocation of factor resources. Consequently, firms with a higher competitive position are better able to mitigate the operational volatility and unpredictability caused by uncertainty, thereby weakening the negative impact of carbon risk on labor investment efficiency.

Following Peress (2010)^50^, The Herfindahl-Hirschman Index (*HHI*) is defined as the sum of the squared ratios of each firm’s operating revenues within an industry to the total industry revenue for each year. The interaction term between *HHI* and *Risk* is included in the regression model 5 to test the moderating effect. The coefficients of these interaction terms capture the differential impact of a firm’s competitive market position on the relationship between carbon risk and labor investment efficiency. Column (1) of Table 7 shows the moderating effect of competitive position on the relationship between carbon risk and labor investment efficiency. The regression coefficient for the interaction term HHI*Risk is −0.102, which is significant at the 10% level, indicating that a higher competitive position mitigates the negative impact of carbon risk on labor investment efficiency.

#### Impact of financing constraints

When enterprises face high carbon risks, they incur significant adjustment costs. These costs are further exacerbated by increased financing constraints. On the one hand, managers must account for the default costs associated with workforce reductions and the sunk costs of prior employee training when making labor reduction decisions; on the other hand, financing constraints prevent them from expanding their workforce in a timely and accurate manner to meet low-carbon emission reduction needs. Therefore, if firms face severe financing constraints, managers will encounter greater limitations in labor investment decisions, such as flexibly adjusting the number of employees based on labor adjustment costs. In other words, increased financing constraints exacerbate the relationship between carbon risk and labor investment efficiency.

This paper employs the *SA* index, a positive indicator, to measure the financing constraints faced by firms. Column (2) of Table 7 shows the impact of the level of financing constraints on the relationship between carbon risk and firms’ labor investment efficiency. As can be seen from the results, the regression coefficient of SA*Risk is 0.176, which is significantly positive at the 5% level. This indicates that the greater the financing constraints, the more difficult it becomes for firms to efficiently allocate and adjust labor factors. In essence, financing constraints exacerbate the negative effect of carbon risk on firms’ labor investment efficiency.

**Table 7:** Moderating effects test.

Variant	(1)	(2)
	Abresid	Abresid
*Risk*	0.075^***^	0.079^***^
	(4.049)	(4.287)
HHI*Risk	−0.102^*^	
	(−1.924)	
*HHI*	0.028	
	(0.796)	
SA*Risk		0.176^**^
		(2.570)
*SA*		−0.121^***^
		(−4.754)
*constant*	0.238^***^	−0.362^**^
	(3.455)	(−2.533)
N	7234	7234
R^2^	0.064	0.067
Controls	Yes	Yes
*Year*	Yes	Yes
*Indcd*	Yes	Yes

### Heterogeneity analysis

#### Inefficient labor investment

Carbon risk makes firms more likely to use the precautionary principle to avoid risk in factor resource allocation decisions. Enterprises following this strategy are more likely to cut labor costs for short-term performance, leading to over-firing or under-hiring, resulting in labor under-investment. Therefore, the impact of carbon risk on labor under-investment in enterprises is more pronounced.

According to Jung et al. (2014)^1^, inefficient labor investment can be classified into two types: over-investment and under-investment. When the change in the number of actually employed employees is greater than the change in the number of optimally (expected) employed employees, it indicates over-investment in labor (*Mresid*), measured as the positive residual of the model 1; conversely, it indicates under-investment in labor (*Lresid*), measured as the negative residual of the model 1. The results of the regression test using model 2 for the above two types of samples are shown in Table 8. It can be seen that carbon risk has a dampening effect on labor efficiency both in the labor overinvestment group (*Mresid*) and in the underinvestment group (*Lresid*). Furthermore, the coefficient difference test between groups indicates that the negative impact of carbon risk on labor investment efficiency is more pronounced in the under-investment group.

**Table 8:** Results of the inefficient labor investment test.

Variant	(1)	(2)
	Mresid	Lresid
*Risk*	0.131^***^	0.187^***^
	(2.741)	(4.451)
*constant*	0.593^***^	0.113^*^
	(3.679)	(1.708)
N	3246	3984
R^2^	0.011	0.065
Controls	Yes	Yes
*Year*	Yes	Yes
*Indcd*	Yes	Yes
The P-value for the difference in coefficients between groups	3.215^***^	

Note: The p-value for the difference in coefficients between groups was calculated based on the estimation of the Chow test for the interaction model, as below.

#### Labor-intensity

There is a significant difference in the number of employees required by labor-intensive versus non-labor-intensive firms. Labor-intensive firms require greater investment in labor and are more dependent on labor inputs. At the same time, in responding to the call for low-carbon emission reductions, these firms may need to adopt more environmentally friendly technologies and equipment to reduce carbon emissions. This process of technological upgrading and transformation can result in higher capital investment and training costs, thus inhibiting the efficiency of their labor investment.

Drawing on Liu and Zhao (2019)^51^, the ratio of a firm’s number of employees to its operating revenue is used to measure labor intensity. The median labor intensity of enterprises each year is used to distinguish between lower and higher labor intensity groups and group regressions are conducted. The regression results are shown in Table 9. The regression coefficient of *Risk* is significantly positive in the high labor-intensity group, indicating that carbon risk significantly inhibits the labor investment efficiency of high labor-intensity enterprises.

**Table 9:** Results of the labor-intensity test.

Variant	(1)	(2)
	High labor intensity	Low labor intensity
	Abresid	Abresid
*Risk*	0.145^***^	0.020
	(4.899)	(0.856)
*constant*	−0.054	0.192^**^
	(−0.374)	(2.193)
N	3617	3617
R^2^	0.068	0.072
Controls	Yes	Yes
*Year*	Yes	Yes
*Indcd*	Yes	Yes

#### Pollution level

Higher-polluting firms are more susceptible to pressures from environmental regulations, carbon pricing mechanisms, and brand reputation. They need more resources to meet environmental regulations and carbon emission limits, and to make more environmental investments and improvements, which may result in firms facing additional costs that affect their production and operational efficiency. Meanwhile, such enterprises tend to be more vulnerable to public and consumer criticism. Failure to effectively address carbon emissions and other pollution-related issues can lead to a loss of market share and damage to their brand and reputation, ultimately reducing the efficiency of their labor investments.

Based on the 2008 Classification List of Industries for Environmental Verification of Listed Companies and the 2016 Circular of the General Office of the National Development and Reform Commission on Effectively Doing the Key Work for the Launch of the National Carbon Emission Trading Market, thirteen categories of listed companies in high-energy-consuming and heavily-polluting industries—such as petrochemicals, chemicals, building materials, iron and steel, non-ferrous metals, papermaking, electric power, aviation, extractive industry, fermentation, textiles, breweries, and pharmaceuticals—are identified as high-pollution enterprises. The group test results are shown in Table 10, indicating that carbon risk inhibits labor efficiency in both high-pollution and low-pollution groups. The coefficient difference test results show that carbon risk has a greater inhibitory effect on labor investment efficiency in enterprises with a higher degree of pollution.

**Table 10:** Results of the pollution level group test.

Variant	(1)	(2)
	High-pollution group	Low-pollution group
	Abresid	Abresid
*Risk*	0.107^***^	0.059^***^
	(3.049)	(2.663)
*constant*	0.110	0.332^***^
	(−1.007)	(3.623)
N	2557	4677
R^2^	0.064	0.069
Controls	Yes	Yes
*Year*	Yes	Yes
*Indcd*	Yes	Yes
The P-value for the difference in coefficients between groups	3.306^***^	

#### Enterprise technology

The impact of carbon risk on labor investment efficiency may vary for firms with different levels of technology. As societal concern for environmental protection and sustainable development increases, non-high-tech firms may face greater market competition and brand image pressure. Consumers and investors may be more inclined to support firms that adopt cleaner technologies and green production methods. In the meantime, compared to their high-tech counterparts, non-high-tech enterprises rely more on traditional, carbon-intensive technologies and methods in their production and operations, which release large amounts of carbon emissions. The introduction of cleaner, low-carbon technologies may require greater investment and lead to production disruptions or adjustments due to the complexity of technological upgrades. Since they rely more on energy and raw materials, increased carbon costs may directly impact production costs, creating a disincentive for firms to invest in labor efficiency.

The total sample is divided into high-tech enterprise groups and non-high-tech enterprise groups for regression, and the results are shown in Table 11. It can be seen that in the sample group of non-high-tech enterprises, the regression coefficient of *Risk* is significantly positive. The findings show that carbon risk has a significant inhibition effect on the labor investment efficiency of non-high-tech enterprises.

**Table 11:** Results of the enterprise technology test.

Variant	(1)	(2)
	High-tech enterprises	Non-high-tech enterprises
	Abresid	Abresid
*Risk*	0.029	0.106^***^
	(0.981)	(4.385)
*constant*	0.294^***^	0.308^***^
	(2.791)	(2.674)
N	4604	2630
R^2^	0.069	0.073
*Controls*	Yes	Yes
*Year*	Yes	Yes
*Indcd*	Yes	Yes

In 2021, the carbon risk level measurements for the sample firms, China Railway Assembly and Industrial Fortune, are identical. However, China Railway Assembly belongs to the heavy pollution industry, is a non-high-tech enterprise, and has high labor intensity, with a labor investment efficiency value of −0.397, classifying it as a labor underinvestment enterprise. Industrial Fortune belongs to the non-heavy pollution industry, is a high-tech enterprise, and has low labor intensity, with a labor investment efficiency value of 0.059, classifying it as a labor over-investment enterprise. This indicates that the inhibitory effect of carbon risk on labor investment efficiency is more pronounced in labor underinvestment enterprises, high labor-intensity enterprises, high-pollution enterprises, and non-high-tech enterprises.

## Conclusions and implications

Using data from China’s A-share listed corporations from 2012 to 2021, this study empirically investigates the impact of corporate carbon risk on labor investment efficiency. Carbon risk reduces corporate labor investment efficiency, according to the findings, which hold up after several robustness tests and corrections for endogeneity issues. The primary mechanisms through which carbon risk influences labor investment efficiency are identified as increased environmental uncertainty, exacerbated agency problems, and reduced managerial capabilities. According to the moderating effects test, the inhibitory effect of carbon risk on firms’ labor investment efficiency is more pronounced when firms have a lower competitive position in the industry and face greater financing constraints. Additionally, the inhibitory effect is more pronounced when firms underinvest in labor, are highly labor-intensive, are heavily polluting, and are non-high-tech.

The study offers the following policy recommendations based on these findings:

First, companies must take proactive measures to address carbon risks. As China’s “dual-carbon” strategy is implemented, the severity of carbon regulation is increasing, and the carbon risk faced by high-carbon companies is also rising. The findings indicate that carbon risk negatively impacts the distribution of enterprise factor resources, including labor. Enterprises must establish and continuously improve an internal governance and management mechanism compatible with low-carbon emission reduction to minimize the negative impacts of carbon risk. This is essential for achieving good business performance and maintaining a competitive advantage in the market.

Second, managers should respond appropriately to market changes by adjusting labor investments. Labor investment encompasses various components, such as hiring, training, and discharging employees. Managers are responsible for coordinating resource allocation among these aspects based on market conditions. The study concludes that carbon risk heightens environmental uncertainty. Therefore, businesses should improve their governance mechanisms. This will help curb managers’ self-interested behavior, allowing them to create more value for the company. Additionally, managers should improve their professional capabilities. In today’s information-driven economy, the ability to fully utilize information is crucial for managers. Managers must use a wide variety of information to make firm management decisions, continuously increase labor investment efficiency, and boost market competitiveness in an ever-changing environment.

Third, to enhance the effectiveness of labor distribution, we will adhere to enterprise-specific regulations. Improving the quality of factors and their allocation efficiency, as well as stimulating changes in quality, efficiency, and power in economic development, is essential to accelerating the progress of China’s socialist market economic system. Furthermore, the research findings indicate that the inhibitory effect of carbon risk on labor investment efficiency varies depending on the firm’s competitive market position, the degree of financing constraints, and the type of labor investment inefficiency. Companies focusing on the effectiveness of their personnel investment should closely integrate their characteristics to facilitate the implementation of individualized strategies. Such decisions will provide a solid foundation for the robust development of the macro economy and the stable operation of micro-enterprises.

Fourth, enterprises may manage carbon risk scientifically while improving labor investment efficiency. Firms should strengthen carbon risk management, enhance their awareness of environmental responsibility, and shift from “passive disclosure” to “active disclosure”, ensuring the transparent release of relevant environmental data. In addressing the challenges posed by carbon risk, enterprises must adjust their governance mechanisms, management models, and workforce strategies to ensure strong business performance and maintain a competitive edge. Moreover, enterprises should refine their corporate management systems, adapt their business strategies proactively, and pursue low-carbon transformation. By responding swiftly to external changes, firms should adjust their labor investment and personnel management strategies in line with market dynamics, thereby fostering green and sustainable development.

A limitation of this paper is that, despite accounting for a wide range of firm characteristics and industry factors, it does not incorporate the influence of macroeconomic policies and all stakeholders. Future research could address this gap by examining macro-level policies, such as the stringency of environmental regulations, as well as internal and external monitoring mechanisms, such as media scrutiny, audits conducted by accounting firms, and internal audit practices. Additionally, further studies could explore the role of managerial characteristics, such as gender and educational background, in influencing the relationship between carbon risk and labor investment efficiency.

## Data Availability

The data used are based on the annual reports of listed companies, CSR reports, company websites, and environmental department websites. Additional data from CSMAR database：https://www.csmar.com/channels/31.html.
